# A Hybrid OFDM-TDM Architecture with Decentralized Dynamic Bandwidth Allocation for PONs

**DOI:** 10.1155/2013/561984

**Published:** 2013-09-30

**Authors:** Taner Cevik

**Affiliations:** Computer Engineering Department, Fatih University, 34500 Istanbul, Turkey

## Abstract

One of the major challenges of passive optical networks is to achieve a fair arbitration mechanism that will prevent possible collisions from occurring at the upstream channel when multiple users attempt to access the common fiber at the same time. Therefore, in this study we mainly focus on fair bandwidth allocation among users, and present a hybrid Orthogonal Frequency Division Multiplexed/Time Division Multiplexed architecture with a dynamic bandwidth allocation scheme that provides satisfying service qualities to the users depending on their varying bandwidth requirements. Unnecessary delays in centralized schemes occurring during bandwidth assignment stage are eliminated by utilizing a decentralized approach. Instead of sending bandwidth demands to the optical line terminal (OLT) which is the only competent authority, each optical network unit (ONU) runs the same bandwidth demand determination algorithm. ONUs inform each other via signaling channel about the status of their queues. This information is fed to the bandwidth determination algorithm which is run by each ONU in a distributed manner. Furthermore, Light Load Penalty, which is a phenomenon in optical communications, is mitigated by limiting the amount of bandwidth that an ONU can demand.

## 1. Introduction

In parallel with the improvements occurring at the backbone side, more sophisticated and bandwidth harvesting user demands have emerged lately. In spite of this mutual capacity and demand increase, the portion residing between the user and the backbone which is called the last (first) mile remains as the bottleneck. The recent technology employed in the last mile is the DSL technology. However, this solution will soon not be able to fulfill the bandwidth requirements of the users running multimedia and real time services such as video conferencing [1, 2]. 6 Mbits/s bandwidth per user was satisfying for all users previously. However, by the rapid progress of multimedia applications offered via Internet such as High Definition TV, existing resources have begun not to meet the needs of some users. These users running bandwidth harvesting applications needed broadband access capacity in order not to suffer delays and experience low-quality performance. Furthermore, due to the improvements in optical signal processing and devices, deployment of optical architectures in the last mile is not as costly as before [3]. 

In order to remedy that bandwidth bottleneck, optical fiber has been deployed instead of traditional copper cable in the last mile. This architecture is called Fiber-to-the-x (FTTx). This appellation differs according to the distance between the end user and the optical fiber [4].

Besides serving the broadband bandwidth to the users, achieving it in lower costs is another challenge to overcome. PONs are considered as the premising solution for that aforementioned challenge by means of their low operational costs, longevity, and huge capacity [5]. In addition to conventional Internet, CATV, and multimedia data, it also supports wireless data such as 3G and 4G [6]. 

All the elements assigned on the network between source and destination, are passive which means no electrical supply is needed [7]. By means of a splitter, fewer number of fibers are deployed, thereby cost and complexity are reduced. On the contrary, in Active Optical Networks (AON), an active curb switch is employed instead of a passive splitter/combiner. That requires electrical supply, physical preservation, and administration of the switch. However, passive splitter just replicates the incoming signal without performing any operations on it. In another type of optical network called point-to-point Optical Networks, individual fibers are deployed for each subscriber. That is, each user is connected to the central office through the fiber assigned specifically for it which is a very cumbersome and expensive solution. 

PONs roughly comprise three elements. The main component of a PON is the Optical Line Terminal (OLT) device that is placed at the local exchange part (Central Office). OLT is responsible for signal conversion between optical and electrical plane. Furthermore, it coordinates multiple access of ONUs to the common fiber towards upstream direction [8]. Another component is the Optical Network Unit (ONU) which is located at the user side and serves as an interface between the OLT and the user domain. ONUs are usually assigned for every user premise such as a curb or a building. ONUs perform the conversion of optical signals into electrical domain and vice versa. Moreover, they schedule users, thereby provide efficient service quality, fair bandwidth usage and better channel utilization. The last important element of a PON is the Optical Splitter/Combiner that is charged to split the optical signal incoming from OLT into several subsignals and distribute them via the fibers connected to the ONUs placed at the premises side. 

ATM is defined as a standard layer 2 protocol of PONs by the Full Service Access Network (FSAN) in the hope that ATM networks will dominate the market. However, Ethernet has won a great victory against ATM and gained great acceptance especially at the user side of PONs. In ATM Passive Optical Networks (APONs), whole IP datagram is segmented into fixed size ATM cells. If one of the cells is dropped or disrupted somehow, all remaining cells comprising the whole IP datagram have to be resent again, even though they arrive to the destination without any corruption. Moreover, the IP datagram cannot be fragmented. That is to say, when an IP datagram is segmented into ATM cells and the remaining part does not contain enough data to fill out completely an ATM cell, empty bits of the ATM cell will be padded which brings an extra overhead. Also, failing to be an inexpensive technology is another important factor for ATM not being considered at the user side of PONs. Due to above-mentioned reasons, APONs have not gained the targeted interest [9].

Ethernet PON (EPON) is an inexpensive, ubiquitous technology that is interoperable with the legacy equipment while supporting different types of data requiring various service qualities. That makes EPONs much more popular and acceptable in the client side. Depending on the fact that 90% of data traffic emerges from or ends at an Ethernet LAN, it is reasonable of Ethernet to be the standard layer-2 protocol for PONs. 

This paper presents a hybrid OFDM/TDM architecture that provisions enhanced service quality, fair, dynamic bandwidth allocation, and utilization for EPONS. Nearly all dynamic bandwidth allocation algorithms suggested for Time Division Multiplexing (TDM) architectures, offer very close solutions and performances. Furthermore, most of the TDM architectures are centralized. The most attractive solution breaking this uniformity is the method that applies echo mechanism at the splitter, thereby all the ONUs become aware of each other's transmission. A latter solution offered for multiple access is the Wavelength Division Multiplexing (WDM) in which different wavelengths (*λ*_1_, *λ*_2_,…, *λ*_*n*_) are assigned to each ONU. Though it is a simple solution to implement, since it requires tunable receiver arrays at the OLT, it is not cost effective. Orthogonal Frequency Division Multiplexing (OFDM) which is a popular wireless broadband access technology has been considered lately to be employed in PONs. By utilizing the penetrating performance of OFDM mechanism, we present a decentralizing dynamic bandwidth allocation algorithm by means of using echo mechanism in the splitter. 

The remainder of this paper is presented as follows.  Section 2 summarizes the multiple access schemes and dynamic bandwidth allocation algorithms utilized in EPONs; Section 3 describes the architecture we propose; in Section 4, simulation results are depicted and the last section concludes the paper.

## 2. Scheduling and Dynamic Bandwidth Allocation

In EPON, communication channel is divided into two subchannels, upstream and downstream. In the downstream direction, PON acts as a point-to-multipoint network. Data sent by the OLT is broadcasted to all ONUs by means of the splitter. ONUs getting data look at the address of the packets whether the designated subscriber belongs to their domain or not. If it is not, packets are discarded. Encryption is applied in order to prevent eavesdropping. Since broadcast mechanism is employed, problems like multiple accessing in the upstream do not occur. On the other hand, some challenges arise in the upstream direction. All ONUs share the same fiber and they inherently attempt to access to the common communication channel at the same time. Since it was nearly impossible for ONUs to detect collisions occurring at the OLT, traditional collision detection mechanisms such as Ethernet's CSMA/CD mechanism was previously difficult to be employed in EPONs [10]. However, development and usage of echo splitters have let ONUs detect other ONUs' signals, thereby requiring CSMA/CD to be applied. To this end, different solutions have been suggested and some of these are described generally in this section.

Dynamic Bandwidth Allocation Solutions depend on the multiplexing mechanism employed. Therefore, they are grouped roughly under four headings, TDM, CSMA/CD, WDM, and OFDM solutions.

### 2.1. TDM

**Figure 1:**
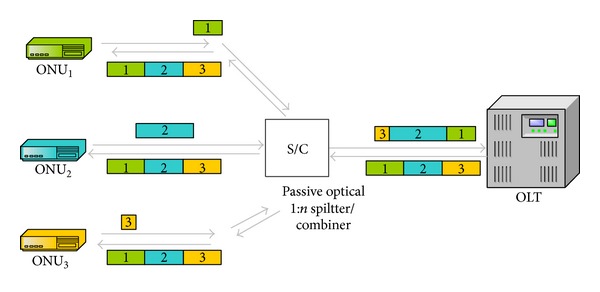
Traffic flow with TDM.

First and the most effective solution offered is the application of TDM in the upstream direction. Time domain is sliced into windows and each ONU is assigned a fix-sized window permanently. This mechanism is very simple and easy to implement. However, it is not an ideal solution due to the bursty nature of the data traffic. Though an ONU does not have any data, the transmission window previously assigned for that ONU cannot serve any other ONU, and the fiber will redundantly remain idle which results in low bandwidth utilization. With the aim of achieving high bandwidth utilization, transmission time windows should be assigned dynamically with varying sizes depending on the burstiness of data [11] as shown in Figure 1. 

In order to provide a better channel utilization, resources should be assigned to demanding ONUs temporarily which is called Dynamic Bandwidth Allocation [12]. Moreover, since time domain is being multiplexed, transmissions of the ONUs must be scheduled depending on different criteria. Some of the proposed solutions employ round-robin mechanism and some of them use adaptive scheduling schemes [13–15]. 

Scheduling is performed in two domains. First one is the Inter-ONU Scheduling, which is the job of organizing the transmissions of multiple ONUs in the system. ONUs inform the OLT about their bandwidth demands. OLT performs required planning operations and replies back to ONUs with their transmission time and length schedules. Another type of scheduling is the Intra-ONU scheduling which is performed by the ONUs in the network. After each ONU gets its GRANT message, obtained bandwidth is apportioned among the users existing in the ONU's domain, depending on their service level agreements or bandwidth demands [16].

All mechanisms suggested for dynamic bandwidth allocation under TDM group are based on multipoint Control Protocol (MPCP) [17]. MPCP is a signaling protocol used by both OLT and ONUs during bandwidth allocation process. MPCP mainly relies on two messages: REPORT and GATE. GATE message is used by the OLT for allocating bandwidth to the ONUs. ONUs send their data together with the REPORT message that carries information about the present situation about the queues of the related ONU. 

Various DBA mechanisms are suggested for the purpose of remedying dynamic bandwidth allocation. As is known, the most popular and fundamental study is IPACT, in which an interleaved polling scheme with an adaptive cycle time is presented. The next ONU is polled, before the data of the previous ONU arrives. In IPACT, for in-band signaling a single wavelength is used for both GRANT message and downstream data transmissions. Besides, they suggest that, in order to prevent some ONUs with larger demands to dominate the whole bandwidth, a maximum window size is defined. If an ONU demands a window size larger than the maximum threshold, the maximum bandwidth it can get is the predefined maximum window size. Demands smaller than the maximum window size will be fulfilled by the OLT. 

Another similar work presented is an adaptive MAC polling protocol suggested by Zheng and Mouftah [11]. This protocol mainly depends on IPACT and the amendment they suggest is defining the ONU that should send its data first by considering their RTTs. 

The problem that emerges during light network loads is the light load penalty identified by Kramer et al. [18]. At the end of every cycle, each ONU puts information about its queues' status into the REPORT message that is attached to the data portion. Obviously, this information denotes the recent bandwidth demand of that ONU. If demand of the ONU is smaller than or equal to the maximum transmission window size, the requested bandwidth is supplied to the ONU by giving the details in the GRANT message. However, until the GRANT message arrives at the ONU, new packets with different priorities inherently may arrive at the ONU from the users located in its domain. The bandwidth granted by the OLT covers the packets arrived previously and the newly arriving high priority packets will suppress the previously arrived lower priority packets. These lower priority packets will have to wait for the next cycle. However, same events may repeat in the next cycle, and the same lower priority packets may suffer unlimited delays and as a result will be dropped. Kramer et al. suggest a two-stage buffer mechanism in which packets arriving at the ONU are placed into different queues depending on their priorities. These queues are called stage 1 queues. Depending on a pulling algorithm, packets are pulled from these differentiated queues and passed to the queue that holds the packets to be transmitted in the next cycle. So, the REPORT message holds information about the status of this stage 2 queue. Newly arriving packets are directly pushed into the stage 1 queues. Thus, they cannot dominate the lower priority packets that have gained transmission grant for the present cycle.

Studies described above are based on the idea of centralized bandwidth allocation and scheduling which is performed by the OLT. ONUs send their bandwidth demands in a REPORT message attached after the data portion. Depending on these requests, OLT allocates bandwidth and organizes the schedule. Despite that, in decentralized solutions, OLT does not have any mission anything about bandwidth allocation and organization process. On the contrary, ONUs decide everything when and how long they will access to the common fiber. This is achieved by means of an echo featured splitter that provides each signal sent by an ONU to be received by the remaining ONUs. In the paper proposed by Sherif et al. [19], time is portioned into frames each of which represents a cycle. A small slice at the beginning of the cycle is divided into small slots that are each assigned to an ONU permanently to announce its bandwidth demand and make reservation. Each ONU broadcasts its demand in its dedicated time slot. After this notification process, all ONUs in the system become aware of the demands of other ONUs. The values obtained from other ONUs are processed by a DBA algorithm. Since every ONU runs the same algorithm, the results produced at each ONU are all consistent. Hence, the schedule becomes definite and every ONU starts its transmission at the time the algorithm identifies.

### 2.2. CSMA/CD

As described at the beginning of this section, CSMA/CD couldn't have been applied in passive optical networks previously, because, it was impossible for an ONU to be aware of another ONUs' transmission. There were two major factors causing ONUs not to be able to realize the signals of other ONUs. First one is that splitters lacked echo (repeater) mechanism. Other reason was that it was very difficult for ONUs to sense a collision occuring at the OLT. By the development of repeater type passive splitters, a signal transmitted by an ONU is echoed at the splitter to all other ONUs. Thus, each ONU realizes an ongoing transmission and does not attempt to send its data in order not to cause a collision which is an obvious application of CSMA/CD. 

RCMA [20] and its extended version FULL-RCMA [21] propose a MAC protocol similar to CSMA/CD. They employ the echo mechanism by means of a passive optical splitter/combiner similar to the 10BASE-FP Ethernet. According to their solution, an ONU, before sending its data, should broadcast a request message. This request message is repeated at the echo splitter and arrives at all other ONUs together with the originating one. When the echoed request message arrives back at the originating ONU, it is discarded, and not evaluated as a collision by means of a special transceiver designed for Ethernet. The request message is used for making reservation in time domain. At that time, all other ONUs become aware of how long the common medium will be busy. Each ONU applies this process and makes its own reservation. Before an ONU broadcasts its requests, it first senses the medium. If it is free to send, then it puts the request message on the way towards the splitter.

### 2.3. WDM

TDM PONs with statistical multiplexing offers low cost network maintenance and administration. However, the common fiber can be used by only a single ONU at a time. Considering bandwidth demands of the clients increase very rapidly nowadays, a promising solution for fulfilling these requirements is the WDM technology. Capacity increase can be achieved in the backbone easily by using WDM. However, employing WDM in the access side has been a much more complicated and expensive challenge. In terms of cost of the devices and maintenance, WDM networks are considered as the next-generation follower of TDM PONs. Thus, hybrid TDM-WDM techniques are being proposed as a smooth adaptation bridge for the recent stage [22].

The most important factor that affects WDMs to be an expensive technology to be applied in the access side is the high cost of the WDM light source. Distributed feedback laser diode used in the backbone is not convenient for the access side [23]. However, promising, low cost WDM light sources are proposed such as Amplified Spontaneous Emission (ASE)-injected Fabry-Perot Laser Diode (FP-LD) [24].

In nondynamic WDM networks, ONUs have a single light source (FP-LD) fixed to a single wavelength. Though this approach seems to be a cost effective solution to implement under light loads; the assigned wavelength will not be fully utilized [25]. Using multiple FP-LDs each fixed to a single wavelength at each ONU is another alternative solution. However, the best case is that each ONU owns a tunable laser that can use any of the upstream channel wavelengths. Though full tunability scheme provides the best performance in terms of bandwidth utilization, it requires a centralized tunable DeMux at OLT which is an expensive solution [26].

### 2.4. OFDM

Another promising technology that is not as costly as WDM for PONs is the Orthogonal Frequency Division Multiplexing. Application of OFDM on PONs has drawn great interest in recent years. By dividing the baseband carrier to the multiple orthogonal subcarriers, significant bandwidth utilization, and enlargements can be achieved [27].

Several research studies [28–30] have been performed about OFDMA PONs. An OFDMA-based 10 Gb/s PON architecture is demonstrated by Qian et al. [31]. In another work [32], a dynamic, asynchronous OFDMA scheme is proposed. Uplink channel is divided into orthogonal subcarriers again. Besides, each ONU has allocated a subchannel permanently dedicated for control messages transmission. Each ONU sends its recent demand to the OLT via that dedicated signaling channel. Depending on these demands arriving from ONUs, OLT allocates the necessary resources to ONUs by means of GRANT messages. In this way, a centralized statistical bandwidth allocation mechanism is achieved.

In the architecture, we propose a statistical subcarrier allocation to each ONU depending on their recent demands. However, instead of charging the OLT for giving this bandwidth allocation decisions, a decentralized approach is employed in which each ONU runs an algorithm that produces the same output at every ONU. Thus, delay suffered because of the REPORT and GRANT signaling messages is alleviated.

## 3. The Proposed Architecture

**Figure 2:**
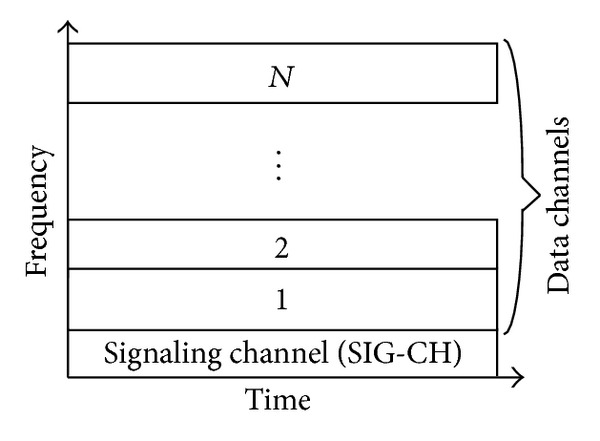
Uplink channel partition in frequency domain.

In traditional TDM PONs, just a single ONU can access to the common channel at a single time. However, by allocating a portion of the capacity to each of the ONUs in the system, multiple of them can access to the common fiber simultaneously. This simultaneous multiple access is achieved by applying OFDMA scheme in our architecture. Uplink channel is apportioned to multiple orthogonal subchannels as shown in Figure 2. 

**Figure 3:**
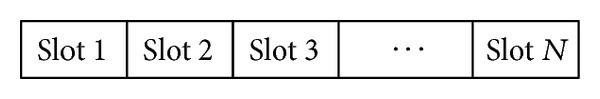
Signaling channel partition in time domain.

Subchannel allocation is not performed centrally by the OLT. As in the previous works, unnecessary delays occur during signaling process. In contrast, a decentralized mechanism is employed. Each ONU in the system runs the same Dynamic Subchannel Allocation algorithm that produces the same output at each ONU. In order to produce the same output, the algorithm has to be applied to the same inputs. These inputs are the bandwidth demands of each ONU in the system. So, these demands should be notified to every ONU. One of the subchannels is assigned for this notification process. Access to the signaling subchannel is achieved in the TDM manner. Hence, signaling subchannel is partitioned into mini-slots in the time domain as depicted in Figure 3, each of which is assigned to an ONU permanently.

### 3.1. System Architecture

**Figure 4:**
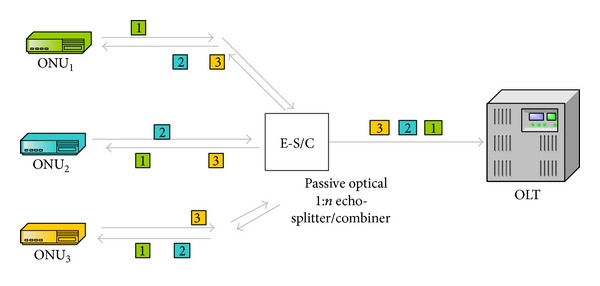
Load notification of ONUs via signaling channel by means of the echo splitter.

Instead of a traditional splitter/combiner, an echo-splitter/combiner [33] is utilized in the system. Thus, each ONU becomes aware of each other's transmissions via a common signaling channel as shown in Figure 4.

**Figure 5:**
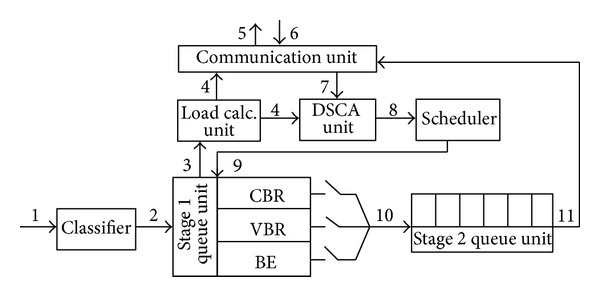
ONU architecture.

Architecture of an ONU in the system is sketched in Figure 5. Numbers and arrows in the figure denote the order and direction of operations, respectively. In order to prevent light-load penalty, two stage queue mechanism is employed in the ONUs. Packets incoming from users (1) are conducted to the appropriate queues by the classifier depending on their types (2). At the beginning of each cycle, Load Calculation Unit (LCU) calculates the total load (3) and passes this information to the Communication Unit (CU) and Dynamic Subchannel Allocation (DSCA) Unit (4). CU broadcasts the load information in its permanently allocated mini-slot period (5) to inform other ONUs about its load. Also, all other ONUs' notification broadcast messages arrive during this load notification stage (6). All incoming load information is processed by the DSCA Unit, and the result which represents the amount of bandwidth that the corresponding ONU gained is conveyed to the Scheduler (8). Scheduler pulls packets from the queues according to the priority management policy without exceeding the bandwidth defined by the DSCA (9). Packets leaving the Stage1 Queue Unit enter to the stage 2 Queue Unit to be sent in the present cycle (10). Lastly, packets ordered in the second queue are forwarded to the CU to be transmitted (11).

### 3.2. Intra-ONU Scheduling and Decentralized Dynamic Bandwidth Allocation

Network traffic is classified as in Diffserv, [34] and packets are pushed into different queues depending on their types. As shown in Figure 5, each ONU maintains three types of queues that each holds different prioritized packets. ConstantBitRate (CBR) stands for Diffserv's Expedited Forwarding (EF) representing services such as voice services that need low delay jitter. VariableBitRate (VBR) symbolizes Diffserv's Assured Forwarding services, such as video that can tolerate some delay and loss. Lastly, BestEffort (BE) is the lowest prioritized services in the hierarchy [35].

Length of each queue that holds different priority packets are passed to the LCU. Bandwidth demand is calculated according to the load present in the queues. The length of high priority queues are the major decisive factor while calculating the load of each ONU. Load of an ONU is calculated by (1):

(1) $$\begin{array}{c} \text{BW}{\text{D}}_{\text{ONU}(i)}=\left(\alpha *\text{LqCBR}\right)+\left(\beta *\text{LqVBR}\right)+\left(\lambda *\text{LqBE}\right). \end{array}$$

In the formula given previously, BWD_ONU(*i*)_ denotes the total load of an ONU, in other words, total bandwidth demand of the ONU. LqCBR, LqVBR, and LqBE represent the lengths of CBR, VBR, and BE queues of ONU(*i*), respectively.

Several simulations are performed for various (*α*, *β*, *γ*) triples. As it will be clarified in the following sections, in order to provide better service qualities for high prioritized packets, larger ratio of (*α*/*β*/*γ*) must be applied. By this way, ONUs with larger CBR queue lengths will possess more bandwidth. 

Total bandwidth demand in the system, which is denoted by TotBwD, is calculated by adding demands of all ONUs in the network:

(2) $$\begin{array}{c} \text{TotBwD}=\overset{N}{\underset{i=1}{\sum }}\text{BW}{\text{D}}_{\text{ONU}(i)}. \end{array}$$

In order to provide fairness, there is a maximum bandwidth value (MaxBw) that an ONU can demand. That is because, if all bandwidth demands of ONUs are satisfied carelessly, ONUs with lower demands will never get a chance to reach to the common fiber. Thus, in order to prevent Light Load Penalty, the amount of bandwidth that an ONU can demand is limited in this way. Calculation of the bandwidth allocated to the ONUs is performed in two stages. In the first stage, demands smaller than MaxBw is satisfied completely. If the value that the ONU demands exceeds MaxBw in the first place, a minimum guaranteed bandwidth (MinGrBw) is allocated to that ONU. In this way, ONUs with larger demands are prevented to dominate the common media and also the ONUs with lower demands get their share fairly. Bandwidth allocation process in the first stage is formulated as follows:

(3) $$\begin{array}{c} {\text{AlBw}}_{\text{ONU}(i)}=\{\begin{array}{cc} \text{BW}{\text{D}}_{\text{ONU}(i)}, & \text{BW}{\text{D}}_{\text{ONU}(i)}\leq \text{MaxBw},\\ \text{MinGrBw}, & {\text{BWD}}_{\text{ONU}(i)}>\text{MaxBw}. \end{array} \end{array}$$

After the bandwidth allocation at the first stage, the remaining bandwidth demand of each ONU is shown by

(4) $$\begin{array}{c} \text{RemBw}{\text{D}}_{\text{ONU}(i)}=\text{BW}{\text{D}}_{\text{ONU}(i)}-\text{AlB}{\text{w}}_{\text{ONU}(i)}. \end{array}$$

Equation (5) represents the total remaining bandwidth demand after the first stage:

(5) $$\begin{array}{c} \text{TotRemBwD}=\overset{N}{\underset{i=1}{\sum }}\text{RemBw}{\text{D}}_{\text{ONU}(i)}. \end{array}$$

The total allocated and remaining available bandwidths after the first stage are calculated, respectively, as follows:

(6) $$\begin{array}{c} \text{TotAlBw}=\overset{N}{\underset{i=1}{\sum }}\text{AlB}{\text{w}}_{\text{ONU}(i)}, \end{array}$$

(7) $$\begin{array}{c} \text{RemAvBw}=\text{TotBw}-\text{TotAlBw}. \end{array}$$

In (7), TotBw and RemAvBw denote the total bandwidth capacity of the network and the remaining available bandwidth after the first stage, respectively. In the second stage, residual bandwidth allocation to the ONUs is performed proportional with their remaining demands as identified by

(8) $$\begin{array}{c} {\text{AlBw}}_{\text{ONU}(i)+}=\;\frac{\text{RemBw}{\text{D}}_{\text{ONU}(i)}}{\text{TotRemBwD}}*\text{RemAvBw}. \end{array}$$

**Figure 6:**
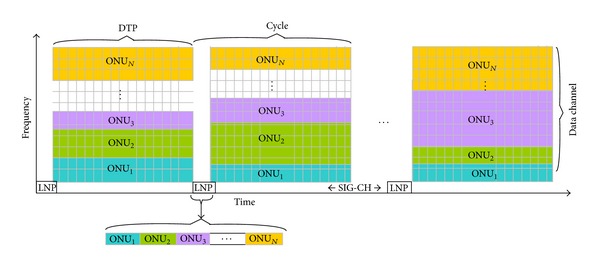
Channel assignment.

Channels are assigned to the ONUs as shown in Figure 6 according to the results calculated by the decentralized dynamic bandwidth allocation algorithm. As represented in Figure 6, time is partitioned into cycles. Each cycle comprises two phases: Load Notification Phase (LDP) and Data Transfer Phase (DTP). During LDP which is negligible compared to the DTP, an ONU broadcasts its load information towards the splitter via the signaling channel in its permanent mini-timeslot. When the broadcasted notification message arrives at the echo-splitter/combiner, it is reflected back towards the other ONUs together with the originating one. By looking at the source id of the message, the originator discards it. Other ONUs check the destination address of the message and after realizing the broadcast address and the type of the message which identifies a notification, their CUs take and pass the message to their DSCA units.

## 4. Performance Evaluation

In this section, we present the results of the simulations performed with different parametric combinations. Simulations are performed on an event-based testbed. We use a PON with 10 ONUs connected in a stare-shape topology. The uplink channel data rate between each ONU and the splitter is assumed as 1 Gb/s with a total capacity of 10 Gb/s. Cycle length in time domain is set to 20 ms. Total bandwidth is divided into 2048 subchannels each with the capacity of 5 Mb/s. Number of users connected to each ONU is assumed to be the same. However, not all of the users connected to an ONU generate packets. Each user generates packets randomly in each cycle. As described previously, three different types of packets are present in the network: CBR, VBR, and BE. CBR packets are generated with the rate of 4.48 Mb/s and 70 Bytes length. Sizes of VBR and BE packets are assumed to be 100 Bytes.

We first identify the impact of the coefficients *α*, *β*, and *λ* on the maximum delay suffered in the system. As described in (1), load of an ONU which actually symbolizes the bandwidth demand of an ONU is calculated according to this triplet (*α*, *β*, *λ*) value. 

**Figure 7:**
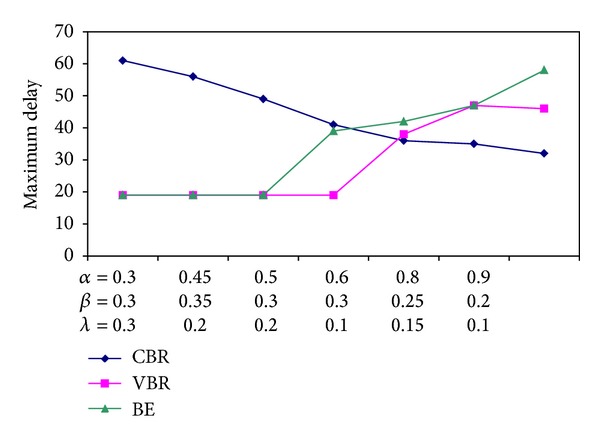
Maximum delay suffered due to different combinations of (*α*, *β*, *λ*) triple.

**Figure 8:**
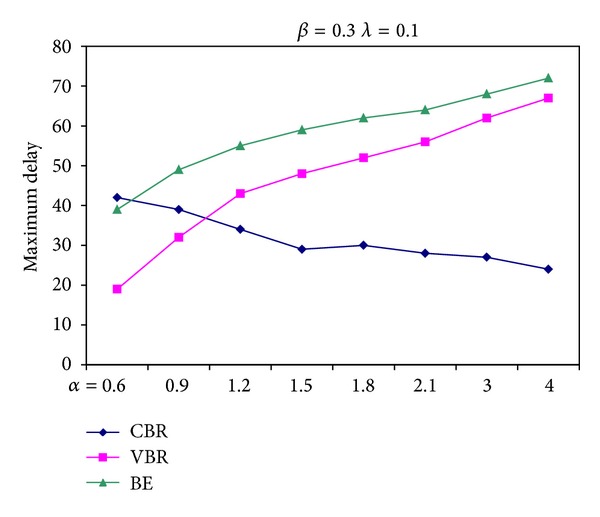
Maximum delay suffered for different values of *α* when *β* and *λ* are kept constant.

Figure 7 shows the maximum delay suffered depending on different (*α*, *β*, *λ*) combinations. As the ratio of *α*/(*β*, *λ*) increases, end-to-end delay of a CBR packet decreases. The reason of the decrease in delay is that *α*/(*β*, *λ*) ratio describes the bandwidth reserved for CBR packets.  Figure 8 also encourages the notion asserted above. It is depicted the change in the maximum delay CBR, VBR, and BE packets suffered due to varying values of *α* when *β* and *λ* are kept constant. 

**Figure 9:**
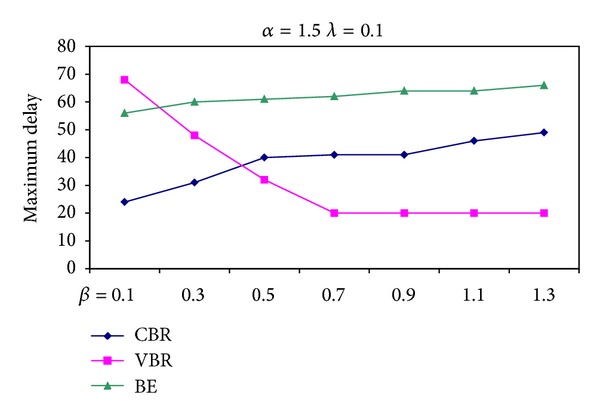
Maximum delay suffered for different values of *β* when *α* and *λ* are kept constant.

Since the parameter *β* describes the weight of VBR packets during bandwidth reservation, as shown in Figure 9, the increase in the value of *β* when *α* and *λ* are kept constant, the delay of VBR packets decreases.

**Figure 10:**
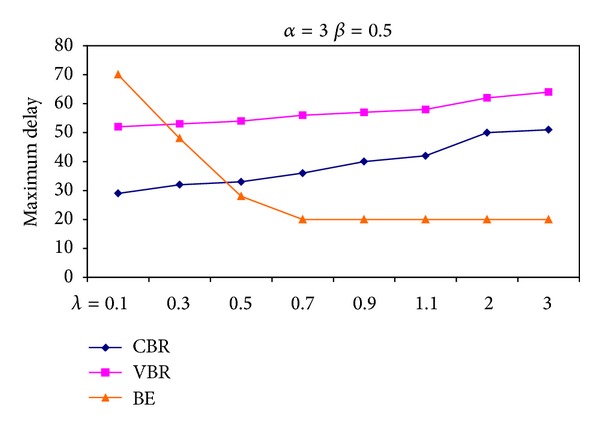
Maximum delay suffered for different values of *λ* when *α* and *β* are kept constant.

The same goes for the BE packets. As presented in Figure 10, when it is intended to prevent the CBR and VBR packets to dominate the BE packets in the system, value of *λ* is increased.

**Figure 11:**
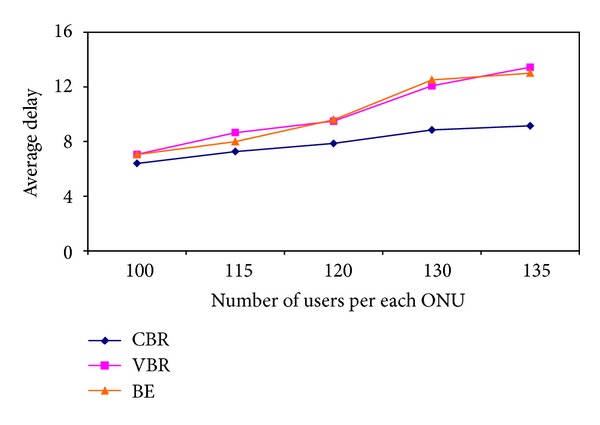
Change in delay due to increase in the number of users.

Another important factor affecting the delay is the network traffic. By the time the number of active users in the system increases, the network load also increases. The change in delay due to the increase in the number of users attached to each ONU is shown in Figure 11. 

**Figure 12:**
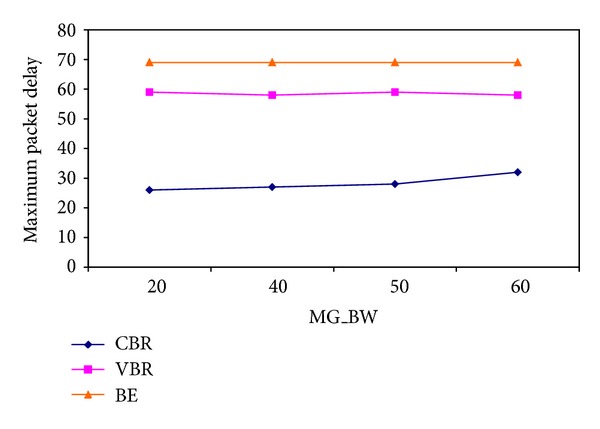
Change in delay due to increase in the amount of minimum guaranteed bandwidth.

As described in the previous section, during bandwidth partition for each ONU, if it is not prevented, an ONU with a higher load can dominate the total capacity. In this case, users of ONUs with lower loads may suffer to significant delays. In order to avoid this situation, all of the ONUs are given some amount of guaranteed bandwidth which is denoted by MinGrBw in (3). The remaining bandwidth is shared rationally between the ONUs of which bandwidth demands are not fulfilled. As clarified in Figure 12, if the amount of minimum guaranteed bandwidth is increased, the delay that the CBR packets suffer also increases.

## 5. Conclusion

This paper proposes a hybrid OFDM-TDM architecture with a statistical bandwidth allocation scheme. Traditional centralized structures mostly apply TDM mechanism as the multiple access scheme. With TDM, just a single ONU owns the common shared medium at a time. However, apportioning the available resources among the beneficiaries yields a much more effective and fair utilization. WDM is an alternative and promising solution for this purpose. Though it is an applicable technology in the backbone, it is not cost effective in the access side because it requires a centralized tunable DeMux in OLT which is an expensive solution. One of the latest prominent solutions for PONs is the application of OFDM in the access side. OFDM provides better shared resource access with lower costs. In this paper, TDM is employed as the accompanying multiple access scheme for coordinating the bandwidth apportioned among the users that is assigned by OFDM. Besides, by applying a decentralized architecture, redundant delays suffered because of the signaling between the ONUs and the OLT are prevented. After a short notification stage, each ONU becomes aware of the loads of other ONUs in the system. Load calculation is performed according to the weights of CBR, VBR, and BE packets in the total load. Hence, high priority packets and ONUs dominating the resources are prevented. Allocating resources dynamically in each cycle according to the demands of ONUs provides better resource utilization.

 For future work, a performance comparison by employing multiple OLTs in the system will be added.
